# A Novel Lid-Covering Peptide Inhibitor of Nicotinic Acetylcholine Receptors Derived from αD-Conotoxin GeXXA

**DOI:** 10.3390/md15060164

**Published:** 2017-06-05

**Authors:** Longjin Yang, Han-Shen Tae, Zhou Fan, Xiaoxia Shao, Shaoqiong Xu, Suwen Zhao, David J. Adams, Chunguang Wang

**Affiliations:** 1 Department of Central Laboratory, Shanghai 10th People’s Hospital, School of Life Sciences and Technology, Tongji University, Shanghai 200092, China; yanglongjin2010@126.com (L.Y.); shxx@tongji.edu.cn (X.S.); xsq1201@163.com (S.X.); 2 Illawarra Health and Medical Research Institute (IHMRI), University of Wollongong, Wollongong, NSW 2522, Australia; hstae@uow.edu.au; 3 iHuman Institute, ShanghaiTech University, Shanghai 201210, China; fanzhou@shanghaitech.edu.cn (Z.F.); zhaosw@shanghaitech.edu.cn (S.Z.); 4 School of Life Science and Technology, ShanghaiTech University, Shanghai 201219, China

**Keywords:** nAChR, conotoxin, αD-GeXXA, NTD, lid-covering

## Abstract

Nicotinic acetylcholine receptors (nAChRs) play a fundamental role in nervous signal transmission, therefore various antagonists and agonists are highly desired to explore the structure and function of nAChRs. Recently, a novel dimeric αD-conotoxin GeXXA was identified to inhibit nAChRs by binding at the top surface of the receptors, and the monomeric C-terminal domain (CTD) of αD-GeXXA retains some inhibitory activity. In this study, the internal dimeric N-terminal domain (NTD) of this conopeptide was further investigated. We first developed a regio-selective protection strategy to chemically prepare the anti-parallel dimeric NTD, and found that the isolated NTD part of GeXXA possesses the nAChR-inhibitory activity, the subtype-dependence of which implies a preferred binding of NTD to the β subunits of nAChR. Deletion of the NTD N-terminal residues did not affect the activity of NTD, indicating that the N-terminus is not involved in the interaction with nAChRs. By optimizing the sequence of NTD, we obtained a fully active single-chain cyclic NTD, based on which 4 Arg residues were found to interact with nAChRs. These results demonstrate that the NTD part of αD-GeXXA is a “lid-covering” nAChR inhibitor, displaying a novel inhibitory mechanism distinct from other allosteric ligands of nAChRs.

## 1. Introduction

Nicotinic acetylcholine receptors (nAChRs) are a fundamental family of pentameric ligand-gated ion channels widely distributed and expressed in the nervous system and non-neuronal cells. In the nervous system, binding of the endogenous neurotransmitter acetylcholine (ACh) onto the nAChR extracellular domain opens the central transmembrane cation channel, leading to depolarization of the postsynaptic neurons or muscle fibers [1]. Dysfunctional nAChRs have been implicated in various neuronal disorders such as Parkinson’s disease and myasthenia gravis [2,3,4]. In addition, as the natural target of nicotine, nAChRs are also the main focus of clinical therapy for smoking cessation [5]. As a consequence, numerous efforts have been made to develop nAChR ligands (agonists, antagonists, and modulators), both for the structural and functional understanding of nAChRs and for potential drug development.

Natural neurotoxins have long been a major reservoir for the identification of different nAChR-targeting ligands, with the most well-studied being the krait α-bungarotoxin since the 1960s and more recently α-conotoxins from the *Conus* sea snails, which bind to the endogenous ACh orthosteric binding site [6,7,8]. In addition, a variety of small allosteric ligands bind to other sites on nAChRs including the pocket beneath the top helix of the extracellular domain, the subunit interface of the extracellular domain, within the ion channel, and the transmembrane domain [9,10,11]. The remarkable diversity of nAChR ligand binding sites suggests that the opening of nAChRs involves global conformational changes and that novel ligands with distinct binding site will potentially provide new understanding on the structure and function of nAChRs.

Conotoxins are a mixture of peptide neurotoxins produced by marine cone snails, targeting different ion channels and neurotransmitter receptors in the nervous system [12]. Due to their remarkable structural and functional diversity, some conotoxin components have satisfying specificity and potency, and consequently, great potential for therapeutic applications. The first FDA-approved conotoxin is ω-MVIIA (commercially named Zinonotide or Prialt), a selective N-type Ca^2+^ channel blocker with analgesic activity [13]. A series of other conotoxins are currently in the development pipeline [14,15,16,17].

**Figure 1:**
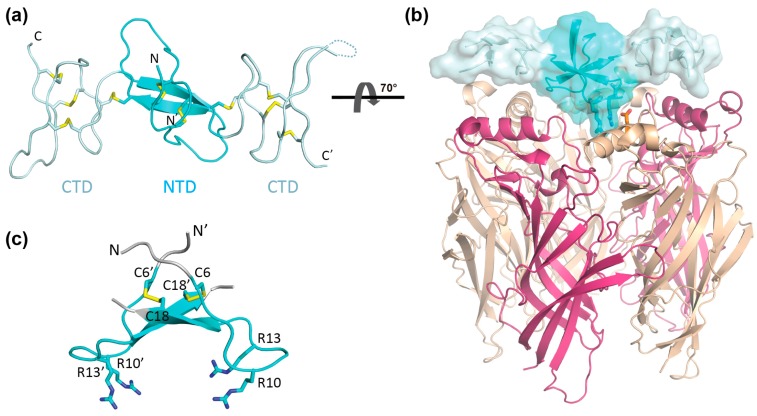
Structure of αD-GeXXA and putative orientation when bound to nicotinic acetylcholine receptors (nAChR). (**a**) Crystal structure of αD-GeXXA (PDB 4X9Z) [18] is shown in cartoon model. Ten disulfide bonds are shown as yellow sticks. The N-terminal domain (NTD) part is colored cyan, whereas the two C-terminal domains (CTDs) are colored pale cyan. (**b**) The putative binding manner of αD-GeXXA onto nAChR. The NTD and CTDs of αD-GeXXA are colored cyan and pale cyan, respectively. The only crystal structure of nAChR currently available (α4β2 subtype, PDB 5KXI, [19]) is used to show the nAChR (pink: α4 subunit; wheat: β2 subunit). For clarity, only the extracellular domains of nAChR are shown. The side chains of putative binding residues, two Arg residues of NTD and an Asp13 residue of a β2 subunit, are shown in stick model. (**c**) Close-up structure of the αD-GeXXA NTD. The terminal residues that are deleted in short NTD are colored gray. The side chains of four downward-facing Arg residues are shown as sticks. Figures are generated using Pymol.

Recently, we described a new nAChR-targeting conopeptide, αD-conotoxin GeXXA, from the venom of *Conus generalis* and revealed that this dimeric peptide toxin exerts its inhibitory effect by binding to the upper surface of the nAChRs [18]. The crystal structure of αD-GeXXA reveals that this dimeric toxin is composed of two C-terminal domains (CTD) joined by an anti-parallel dimeric N-terminal domain (NTD) (Figure 1). The monomeric CTD retains weak nAChR inhibitory activity, putatively by binding at the top surface between two nAChR subunits [18]. This binding mode places the internal dimeric NTD covering the center of the nAChR top surface (Figure 1b), which raises the possibility that the NTD part of αD-GeXXA may also contribute to the interaction with nAChRs. In addition, the orientation of αD-GeXXA when bound onto nAChRs remains elusive, which hinders better understanding of its mechanism of action.

To address these questions, we first chemically prepared αD-GeXXA NTD, and showed that it inhibited ACh-evoked currents mediated by nAChRs. We then prepared the truncated NTD, with the N-terminal and C-terminal residues deleted. This short NTD (sNTD) exhibits similar inhibitory activity as the full-length NTD, indicating that the N-terminus of this toxin is not involved in the interaction with nAChRs, thus clarifying the orientation of αD-GeXXA when bound to nAChRs. In order to simplify the preparative procedure of NTD, we designed a single chain peptide cyclized through one terminal disulfide bond (cNTD) and confirmed that the cNTD retains similar inhibitory activity as the original NTD. Using the active cNTD as template, 4 Arg residues were found to be critical for nAChR inhibitory activity. These results demonstrate that the isolated NTD part of αD-GeXXA can function in a “lid-covering” mode to inhibit the opening of the nAChR-channel. In addition, the establishment of the easily-prepared cNTD paves the way for further optimization and mechanism study of this novel nAChR inhibitor.

## 2. Results

### 2.1. Preparation of αD-GeXXA NTD

**Figure 2:**
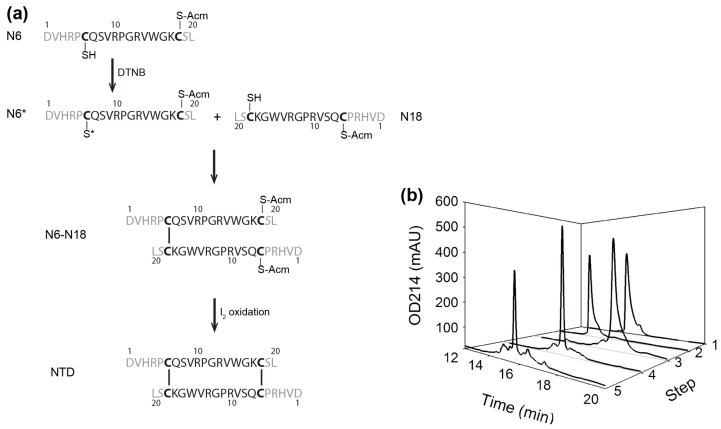
Preparation of the dimeric NTD. (**a**) Preparation procedure of the anti-parallel dimeric NTD from single chain N6 and N18 peptides. In the sequences of N6 and N18, the residues deleted in short NTD are colored gray. (**b**) Peptide HPLC profile of each preparation step. 1: N18 peptide; 2: N6 peptide; 3: 5,5′-dithiobis-(2-nitrobenzoic acid) (DTNB)-activated N6* peptide; 4: dimeric N6-N18; 5: final NTD. The elution gradient for these 5 products is 20–35% Buffer B in 0–15 min.

A regio-protection strategy was utilized to prepare the NTD that is homo-dimerized through two anti-parallel inter-chain disulfide bonds between residues Cys6 and Cys18 (Figure 1c and Figure 2). To ensure the proper pairing of the disulfide bonds, we synthesized two differently protected peptides corresponding to residues 1–20 of αD-GeXXA, with Cys18 of one peptide and Cys6 of the second peptide protected with acetamidomethyl (Acm) group. It is worth noting that, in this study, Cys19 that forms an intra-chain disulfide bond with Cys28 in the full-length αD-GeXXA was mutated into Ser in NTD peptides, to avoid the interference of disulfide pairing by this additional Cys.

While the first disulfide bond should be formed between Cys6 of the first peptide (N6) and Cys18 of the second peptide (N18), mixing these two peptides together, however, would give rise to three possible products: N6 homodimer, N18 homodimer, and N6-N18 heterodimer. In order to ensure that only the heterodimer is formed, we took advantage of the thiol-activating property of DTNB (5,5′-dithiobis-(2-nitrobenzoic acid)) and modified Cys6 of N6 peptide with this reagent. Then, N6 peptide with activated Cys6 (N6*) was mixed with N18 peptide with unmodified Cys18, to specifically form the first inter-chain disulfide bond. Subsequently, the second disulfide bond (between Cys18 of N6 peptide and Cys6 of N18 peptide) was formed under the oxidation of iodine (Figure 2). The reaction and the product purity of each step were confirmed by mass spectrometry (Table S1).

### 2.2. nAChR-Inhibitory Activity of NTD

**Figure 3:**
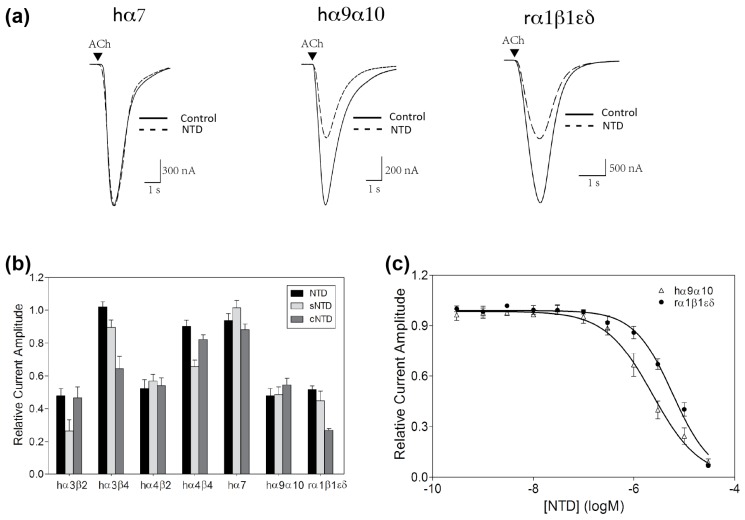
Inhibition of nAChR subtypes by αD-GeXXA NTD. (**a**) Superimposed acetylcholine (ACh)-evoked currents mediated by hα7, hα9α10 and rα1β1εδ nAChR subtypes in the absence (solid lines) and presence (dashed lines) of 5 μM NTD. (**b**) Relative current amplitude of different nAChR subtypes obtained in the presence of 5 μM NTD, sNTD, and cNTD in comparison to the absence of the NTD peptides (mean ± SEM, *n* = 3–8). (**c**) Concentration–response curves of NTD on hα9α10 and rα1β1εδ nAChRs expressed in *X. laevis* oocytes (*n* = 5–8 oocytes for each data point). The IC_50_ values of NTD at hα9α10 and rα1β1εδ nAChR subtypes are 2.33 μM and 5.88 μM, respectively.

To determine whether the NTD retains nAChR inhibitory activity of the full-length αD-GeXXA, the functional activity of the peptide (5 μM) was tested against ACh-evoked currents mediated by different nAChR subtypes heterologously expressed in *Xenopus laevis* oocytes. While the ACh-evoked currents of human (h) α3β4, α4β4, and α7 nAChRs were not markedly affected, 5 μM NTD reduced the ACh-evoked current amplitude at hα3β2, hα4β2, hα9α10, and rodent (r) α1β1εδ nAChRs by approximately 50% (Figure 3a,b).

Concentration-dependent activity of NTD at hα9α10 nAChR, the subtype most preferred by the full-length αD-GeXXA [18], was determined, giving a half-maximal inhibitory concentration (IC_50_) of 2.33 μM (Figure 3c, Table 1). The potency of the NTD is similar to the monomeric αD-GeXXA CTD (2.02 μM), suggesting that NTD also contributes to the high potency of αD-GeXXA (28 nM) at inhibiting the hα9α10 nAChR subtype [18].

In comparison, the IC_50_ of NTD at the rodent muscle nAChR subtype was 5.88 μM (Figure 3c, Table 1). At this subtype, the inhibitory activity of the monomeric CTD was too low to be determined [18]. Therefore, it appears that the NTD part rather than the CTDs of αD-GeXXA plays a major role in inhibiting the rodent muscle nAChR.

### 2.3. Preparation and Activity of Truncated NTD

The dimeric αD-GeXXA is postulated to bind onto the top surface of nAChRs, for which there are two possible orientations: with the N-terminus facing upwards (as shown in Figure 1b), or downwards. This can be distinguished by deleting the N-terminal extension residues. However, due to the complex structure of αD-GeXXA with 10 disulfide-bonds per molecule [18], it is difficult to refold the deletion mutations of dimeric αD-GeXXA. Given that the NTD peptide has inhibitory activity, the binding orientation of the toxin can be determined in the context of NTD.

**Figure 4:**
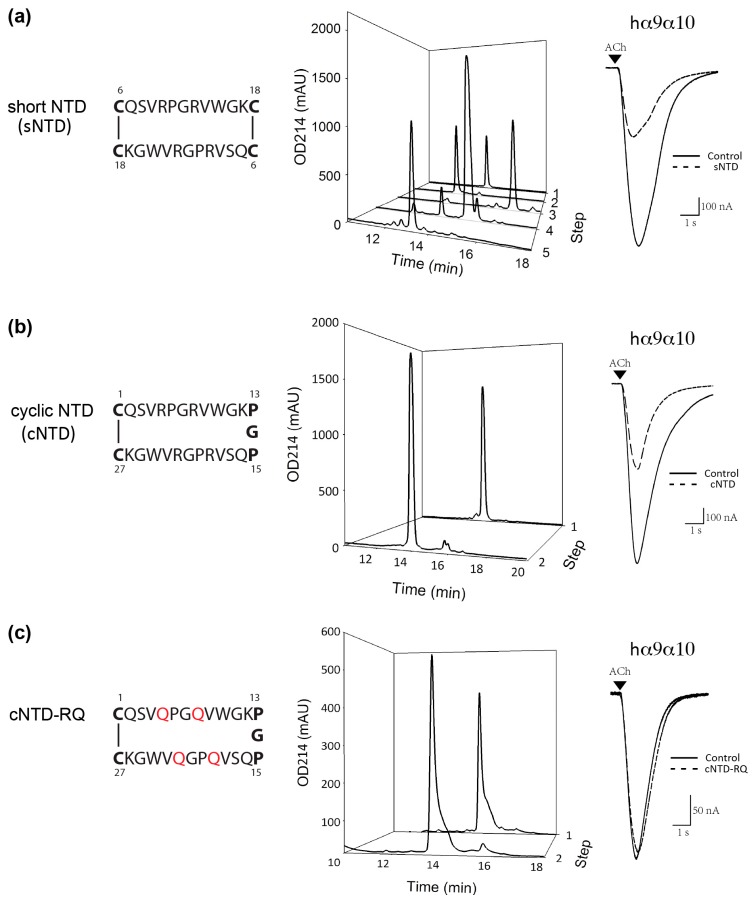
Preparation and functional activity of sNTD, cNTD, and cNTD-RQ, a mutant of cNTD where all the 4 Arg residues are mutated to Gln. (**a**) Left panel: the topological structure of sNTD (Cys residues are highlighted in bold). Middle panel: peptide HPLC profile of each sNTD preparation step. 1: sN18 peptide; 2: sN6 peptide; 3: DTNB-activated sN6* peptide; 4: dimeric sN6-sN18; 5: final sNTD. Right panel: ACh-evoked currents mediated by hα9α10 nAChR in the absence (solid line) and presence (dashed line) of 5 μM sNTD. (**b**) Left panel: the topological structure of cNTD (Cys residues and Pro-Gly-Pro linker are highlighted in bold). Middle panel: peptide HPLC profile of each cNTD preparation step. 1: linear cNTD peptide; 2: final cNTD. Right panel: ACh-evoked currents mediated by hα9α10 nAChR in the absence and presence of 5 μM cNTD. (**c**) Left panel: the topological structure of cNTD-RQ. Four Arg residues are mutated to Gln (orange). Middle panel: peptide HPLC profile of each cNTD-RQ preparation step. 1: linear cNTD-RQ peptide; 2: final cNTD-RQ. Right panel: ACh-evoked currents mediated by hα9α10 nAChR obtained in the absence and presence of 5 μM cNTD-RQ. The elution gradient for all these peptide HPLC profiles is 20–35% Buffer B in 0–15 min.

Therefore, using the strategy for NTD preparation (Figure 2), we prepared the dimeric short NTD (sNTD), with the N-terminal 5 residues (Asp, Val, His, Arg, and Pro) and C-terminal 2 residues (Ser and Leu) omitted (Figure 4a and Table S1). At 5 μM, sNTD exhibited similar functional activity to NTD (Figure 3b), indicating that the N-terminus of this toxin is not required for the interaction with nAChRs. This also clarifies that αD-GeXXA binds to nAChRs with the N-terminus facing upwards (Figure 1b).

### 2.4. Preparation and Activity of Cyclic NTD

The feasibility of chemically preparing the active dimeric NTD makes further mutagenesis study possible, but the multiple-step procedure (Figure 2) is time-consuming. We sought to simplify this procedure by reducing the number of disulfide bonds, based on the simpler sNTD construct. We noticed from the topological structure of sNTD (Figure 4a) that the dimeric sNTD is a tandem repeat of two sNTD sequences, but linked by one disulfide bond in between, and another disulfide bond to cyclize the two termini. Therefore, we removed the internal disulfide bond by replacing two Cys residues with two Pro residues, and then linked the two repeat peptides with a Gly residue into a single peptide chain. This chemically synthesized single polypeptide chain was conveniently cyclized by oxidizing the only intra-chain disulfide bond to form a cyclic NTD (cNTD) (Figure 4b).

**Figure 5:**
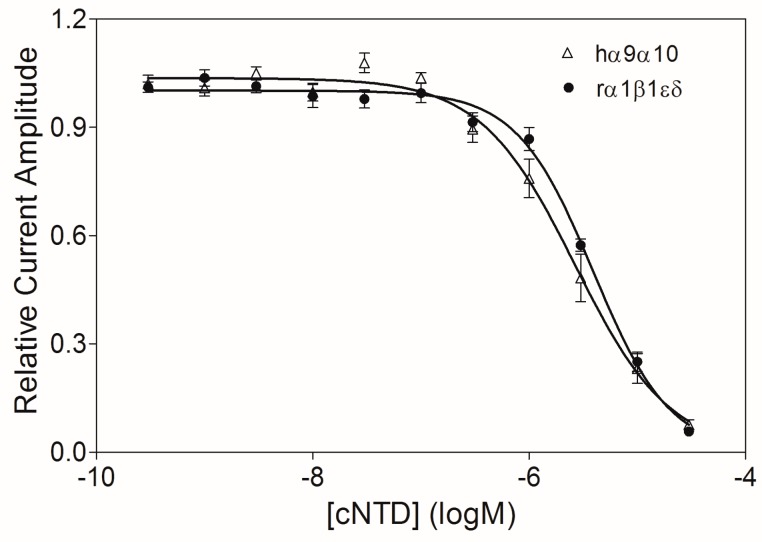
Concentration–response relationships of cNTD at hα9α10 and rα1β1εδ nAChRs expressed in *X. laevis* oocytes (*n* = 5–7 oocytes for each data point). The IC_50_ values of cNTD at hα9α10 and rα1β1εδ nAChR subtypes are 2.66 μM and 3.91 μM, respectively.

As expected, cNTD retained the same nAChR inhibitory profile as the full-length NTD and the truncated sNTD (Figure 3b). We also determined the IC_50_ values of cNTD at hα9α10 and rα1β1εδ nAChRs (Figure 5, Table 1), which are similar to those of the full-length NTD (Figure 3c). The comparable activity of cNTD demonstrates that the artificial PGP (Pro-Gly-Pro) motif satisfyingly adopts the conformation of the two Cys residues linked through an inter-chain disulfide bond, and renders cNTD the same conformation as the dimeric sNTD. Moreover, the one-step oxidation procedure of cNTD makes future mutational study of this peptide easier.

Then, with the binding orientation of NTD clarified, we investigated the downwards-facing residues of NTD for potential nAChR-binding sites. Among them, the downwards-protruding side chains of 4 Arg residues (two Arg10 and Arg13 residues respectively) are prominent (Figure 1c). Indeed, replacement of Arg residues with Gln residues (cNTD-RQ) abolished the inhibition of ACh-evoked currents at the hα9α10 nAChR (Figure 4c), demonstrating the importance of the 4 Arg residues in the interaction with nAChRs.

## 3. Discussion

To date, a number of different conotoxin families have been identified to target nAChRs, including the most extensively studied ACh-competitive α-conotoxins [8]. Other nAChR-targeting conotoxins include the ψ-, αB-, αC-, and αS-families, but their binding sites on nAChRs are currently unknown [20,21,22,23]. In contrast, another superfamily of nAChR-targeting conotoxin, namely αD-conotoxins, was recently identified with a novel mechanism of action by binding at the top surface of nAChRs [18]. Following this discovery, we further demonstrate in this study that only the NTD part of αD-conotoxin GeXXA is a “lid-covering” antagonist of nAChR.

αD-Conotoxins have been identified from various *Conus* species, including *C. generalis* [18], *C. vexillum* [24], *C. capitaneus*, *C. mustelinus*, and *C. miles* [25], and possibly make a significant contribution to their toxicology. The nanomolar potency of αD-conotoxins at nAChRs [18] suggests that understanding their mechanism may be valuable for the development of new nAChR inhibitors. The NTD activity described in this study revealed that, at least for αD-GeXXA, the high potency is a result of the cooperative nAChR-binding of not only the two CTDs but also the NTD part. In addition, sNTD retains the potency of NTD at inhibiting nAChRs, which suggests an N-terminal-upward orientation of the αD-GeXXA when bound to nAChRs (Figure 1b).

The novel “lid-covering” inhibitory mechanism of NTD is clearly different from the typical nAChR-targeting neurotoxins (α-bungarotoxin and α-conotoxins) that bind to the interface between the extracellular domains of two adjacent subunits [26,27]. The “lid-covering” inhibition is also distinct from the pore blockers, as NTD binds to the top surface of the nAChR extracellular domain rather than within the transmembrane ion channel. Because binding of NTD does not fully seal the entrance of the receptor-channel pore (Figure S1), we propose that the mechanism of NTD inhibition at nAChRs is not by simply blocking the cations from entering the channel, but rather by preventing conformational changes of the extracellular domain that are necessary in linking agonist binding to the opening of the transmembrane channel [28]. Although it remains to be tested whether the agonist can bind to the NTD-bound nAChRs, it is likely that the binding of NTD traps the receptor in the resting-closed state.

Another interesting feature of NTD is that it binds only to the N-terminal region of the nAChRs. Different nAChR subunits have highly homologous sequences and structures, but the N-terminal region is less conserved (Figure S2). This suggests that “lid-covering” inhibitors would be advantageous to gain better subtype specificity. Comparing the effects of GeXXA-NTD on α3β2 vs. α4β2 and α3β4 vs. α4β4 (Figure 3b) suggests that the change of α subunit does not make much difference to the inhibitory activity of GeXXA-NTD and that GeXXA-NTD mainly binds to non-α subunits. Similarly, it appears that GeXXA-NTD prefers β2-containing rather than β4-containing nAChR subtypes (Figure 3b). Consistent with the finding that Arg10 and Arg13 residues in GeXXA-NTD are critical for nAChR inhibitory activity (Figure 4c), two more acidic residues (Asp2 and Asp13) are present in the N-terminal α-helix region of the β2 subunit in comparison to β4 subunit (Figure S2). Further study would be required to address whether these acidic residues are indeed the nAChR-contacting points of GeXXA-NTD.

In summary, by exploring the NTD part of αD-conotoxin GeXXA, we revealed a novel “lid-covering” inhibitory mechanism for nAChRs. This reinforces the notion that natural neurotoxins are a valuable reservoir for drug leads or tool reagents. Furthermore, establishment of the activity of the easily-prepared cyclic NTD in this work paves the way for further rational design for new “lid-covering” inhibitors with desired subtype specificity, which would be useful for better understanding of the structure and function of the physiologically fundamental nAChRs.

## 4. Materials and Methods

### 4.1. Preparation of NTD and sNTD

To prepare the anti-parallel dimeric NTD, two peptides (N6 and N18) with the sequence of αD-GeXXA residues Asp1-Leu20 were chemically synthesize (Chinese Peptide, Hangzhou, China). In peptide N6, the thiol group of Cys6 was not protected, whereas the thiol group of Cys18 was protected with acetamidomethyl (Acm) group. In peptide N18, the thiol group of Cys6 was protected with Acm, while the Cys18 side chain was kept free. In both peptides, residue Cys19 was mutated into Ser.

To activate the thiol group of Cys6 in peptide N6, 1 μg/μL of peptide was mixed with 1 mM 5,5'-dithiobis-(2-nitrobenzoic acid) (DTNB) (Bio Basic Inc., Markham, ON, Canada) in 150 mM PBS pH 7.3 and reacted for 15 min at room temperature (20–25 °C) in the dark, then excess DTNB reagent was removed from the activated N6 (N6*). The first disulfide bond between Cys6 of N6 and Cys18 of N18 was formed by mixing 0.4 μg/μL N6* and 0.3 μg/μL N18 in 150 mM PBS pH 7.3, and reacting for 15 min at room temperature. The dimeric N6-N18 was further oxidized with 2 mM iodine in 75% acetic acid, 150 mM HCl in the dark for 10 min to form the second disulfide bond between Cys18 of N6 and Cys6 of N18. The iodine oxidation was quenched with 150 mM ascorbic acid. The synthesized peptides and the reaction product of each step were purified on a Zobax C18 column (250 × 4.6 mm, Agilent, Santa Clara, CA, USA) with an acetonitrile elution gradient using an Agilent 1100 HPLC system. Buffer A for HPLC purification is 0.1% trifluoroacetic acid (TFA) (Merck, Fairfield, OH, USA) in H_2_O and Buffer B is 0.1% TFA in acetonitrile (Duksan Pure Chemicals Co Ltd., Ansan, Korea).

Peptide purity and identity were assessed by Q-trap mass spectrometer (Applied Biosystems, Foster City, CA, USA), using the scan type of Enhanced MS. The apparatus was equipped with a TurboIonSpray source and operated in positive ionization mode.

To prepare sNTD, two peptides (sN6 and sN18) with the sequence of αD-GeXXA residues Cys6-Cys18 were chemically synthesize (Chinese Peptide, Hangzhou, China), and the same strategy and reaction conditions as NTD preparation were used.

### 4.2. Preparation of cNTD and cNTD-RQ

To obtain the cyclic NTD, a linear peptide of 27 residues with two Cys residues at both termini was chemically synthesized (Chinese Peptide, Hangzhou, China). The peptide was dissolved in 400 mM Arginine, 50 mM Tris·HCl pH 8.1 to 0.075 μg/μL concentration and air-oxidized for 24 h at 4 °C, and then purified with acetonitrile elution gradient on HPLC C18 column. The linear peptide of cNTD-RQ was chemically synthesized (GL Biochem Ltd., Shanghai, China) and cyclized under the same conditions as cNTD.

### 4.3. Electrophysiological Recordings from nAChRs Exogenously Expressed in Xenopus Oocytes

Oocyte preparation, RNA preparation, and expression of nAChR subunits in *Xenopus* oocytes were performed as described previously [18]. All procedures were approved by the University of Sydney Animal Ethics Committee. Plasmid constructs of rat (α1, β1 and δ), mouse (ε), and human (h) (α3, α4, α7, α9, α10, β2 and β4) nAChR subunits were linearized for in vitro mRNA synthesis using mMessage mMachine transcription kit (AMBION, Forster City, CA, USA).

Stage V–VI *Xenopus laevis* oocytes were defolliculated with collagenase (Worthington Biochemical Corp., Lakewood, NJ, USA) at room temperature (20–25 °C) for 1 h in OR-2 solution containing (in mM) 82.5 NaCl, 2 KCl, 1 MgCl_2_, and 5 HEPES at pH 7.4. Oocytes were injected with 5 ng cRNA for hα3β2, α3β4, α4β2, α4β4, α7 or rodent (r) α1β1δε nAChRs and 35 ng cRNA for hα9α10 nAChR (concentration confirmed spectrophotometrically and by gel electrophoresis) using glass pipettes. Oocytes were incubated at 18 °C in sterile ND96 solution composed of (in mM) 96 NaCl, 2 KCl, 1 CaCl_2_, 1 MgCl_2_, and 5 HEPES at pH 7.4, supplemented with 5% fetal bovine serum, 50 mg/L gentamicin (GIBCO, Grand Island, NY, USA) and 10,000 U/mL penicillin-streptomycin (GIBCO, Grand Island, NY, USA).

Membrane currents were recorded from oocytes expressing nAChRs at room temperature, using a GeneClamp 500B amplifier and pClamp9 software interface (Molecular Devices, Sunnyvale, CA, USA) in a two-electrode voltage-clamp recording configuration (holding potential −80 mV). Voltage-recording and current-injecting microelectrodes were pulled from GC150T-7.5 borosilicate glass (Harvard Apparatus Ltd., Holliston, MA, USA), giving tip resistances of 0.3–1.5 MΩ when filled with 3 M KCl. Oocytes were perfused with ND96 solution at a rate of 2 mL/min. Oocytes expressing hα9α10 nAChRs were incubated in 100 μM BAPTA-AM ~3 h before recording and perfused with ND115 solution containing (in mM): 115 NaCl, 2.5 KCl, 1.8 CaCl_2_, and 10 HEPES at pH 7.4. Due to the Ca^2+^ permeability of hα9α10 nAChRs, BAPTA-AM incubation was carried out to prevent the activation of *X. laevis* oocyte endogenous calcium-activated chloride channels.

Initially, oocytes were briefly washed with bath solution (ND96/ND115) followed by 3 applications of acetylcholine (ACh) at half-maximal effective concentration (EC_50_) values of 6 μM for hα3β2, hα9α10, and hα4β4 nAChRs, 300 μM for hα3β4, 100 μM ACh for hα7, 3 μM for hα4β2, and 1 μM ACh for rα1β1δε nAChRs. Washout with bath solution was done for 3 min between ACh applications. Oocytes were incubated with peptides for 5 min with the perfusion system turned off, followed by co-application of ACh and peptide with flowing bath solution. All peptide solutions were prepared in ND96/ND115 + 0.1% bovine serum albumin. Peak current amplitude evoked by ACh was measured before and following incubation with peptide in order to determine the effect on specific nAChR subtype. Concentration-dependent response curves for antagonists were fitted by unweighted nonlinear regression to the following logistic equation:

(1) $$E_{x}=E_{\max}X^{n^{H}}/(X^{n^{H}}+{{\mathrm{IC}}_{50}}^{n^{H}})$$

where *E_x_* is the response, *X* is the antagonist concentration, *E*_max_ is the maximal response, *n^H^* is the slope factor, and IC_50_ is the antagonist concentration giving half-maximal response. Concentration-dependent response curve and relative current amplitude bar graph data were pooled (*n* = 3–8 oocytes for each data point) and represented as means ± standard error of the mean (SEM). The IC_50_ was determined from the concentration–response curve and reported with 95% confidence interval (CI). Computation was performed using GraphPad Prism 5 (GraphPad Software, La Jolla, CA, USA).

## Figures and Tables

**Table 1:** Inhibitory activity of αD-GeXXA, CTD, NTD, and cNTD at hα9α10 and rα1β1εδ nAChRs.

Peptide	hα9α10		rα1β1εδ	
	IC_50_ (95% CI)	Hill Slope (*n^H^*)	IC_50_ (95% CI)	Hill Slope (*n^H^*)
αD-GeXXA ^1^	28 nM (22–35)	−1.3	743 nM (606–911)	−1.6
CTD ^1^	2.02 μM (1.82–2.25)	−1.7	- ^2^	- ^2^
NTD	2.33 μM (1.92–2.83)	−0.9	5.88 μM (4.71–7.34)	−1.1
cNTD	2.66 μM (2.15–3.29)	−1.0	3.91 μM (3.35–4.56)	−1.2

^1^ Data from [12]; ^2^ Not detected.

## Supplementary Materials

The following are available online at www.mdpi.com/1660-3397/15/6/164/s1. Table S1: Molecular masses of peptides from NTD and sNTD preparation; Figure S1: Top view of the αD-GeXXA NTD bound onto the top surface of the human α4β2 nAChR subtype; Figure S2: Sequence alignment of the extracellular domain of nAChR subunits studied in this work.
