# Supplementary material for: A Novel Lid-Covering Peptide Inhibitor of Nicotinic Acetylcholine Receptors Derived from αD-Conotoxin GeXXA
Source: Mar Drugs. 2017 Jun 5;15(6):164. doi: 10.3390/md15060164 (PMC5484114; doi:10.3390/md15060164)
Supplement: Supplementary file 1 [file marinedrugs-15-00164-s001.pdf]

Article

A novel lid-covering peptide inhibitor of nicotinic acetycholine receptors derived from αD-conotoxin GeXXA

Longjin Yang [1], Han-Shen Tae [2], Zhou Fan [3,4,5], Xiaoxia Shao [1], Shaoqiong Xu [1], Suwen Zhao [5,6], David J. Adams [2,*] and Chunguang Wang [1,*]

1 Department of Central Laboratory, Shanghai 10th People's Hospital, School of Life Sciences and Technology, Tongji University, Shanghai 200092, China; yanglongjin2010@126.com (L.Y.); shxx@tongji.edu.cn (X.S.); xsq1201@163.com (S.X.)

2 Illawarra Health and Medical Research Institute (IHMRI), University of Wollongong, Wollongong NSW 2522 Australia; hstae@uow.edu.au (H.T.)

3 Key Laboratory of Computational Biology, Max Planck Independent Research Group on Population Genomics, CAS-MPG Partner Institute for Computational Biology, Shanghai Institutes for Biological Sciences, Chinese Academy of Sciences, Shanghai 200031, China; fanzhou@shanghaitech.edu.cn (Z.F.)

4 University of Chinese Academy of Sciences, Beijing 100049, China;

5 School of Life Science and Technology, ShanghaiTech University, Shanghai 201219, China;

6 iHuman Institute, ShanghaiTech University, Shanghai 201210, China; zhaosw@shanghaitech.edu.cn (S.Z.)

* Correspondence: djadams@uow.edu.au (D.J.A.); Tel.: +61-2-4239-2264

* Correspondence: chunguangwang@tongji.edu.cn (C.W.); Tel.: +86-21-6598-4347

Supplementary Materials include:

Supplementary Table S1;
Supplementary Figure S1-S2.

Table S1. Molecular masses of peptides from NTD and sNTD preparation.

Step	Peptide	NTD		sNTD	
		**Theoretical mass**	**Determined mass**	**Theoretical mass**	**Determined mass**
1	N18	2352.65	2353.0	1547.75	1548.0
2	N6	2352.65	2353.0	1547.75	1548.0
3	N6*	2548.83	2549.0	1743.93	1744.0
4	N6-N18	4702.30	4702.0	3092.50	3093.0
5	NTD	4558.30	4558.0	2948.50	2948.0

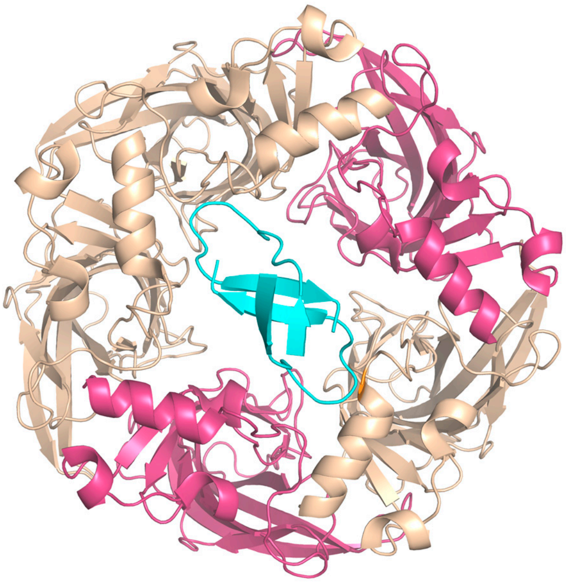

Figure S1: Top view of the αD-GeXXA NTD (cyan) bound onto the top surface of the human α4(pink)β2(wheat) nAChR subtype (PDB 5KXI).

```
rα1    1    --SEHETRLVAKLFKDYSSVVRPVGDHREIVQVTVGLQLIQLINVDEVNQIVTTNVRLKQ 58
hα3    1    --SEAEHRLFERLFEDYNEIIRPVANVSDPVIIHFEVSMSQLVKVDEVNQIMETNLWLKQ 58
hα4    1    --AHAEERLLKKLFSGYNKWSRPVANISDVVLVRFGLSIAQLIDVDEKNQMMTTNVWVKQ 58
hα7    1    --GEFQRKLYKELVKNYNPLERPVANDSQPLTVYFSLSLLQIMDVDEKNQVLTTNIWLQM 58
hα9    1    ADGKYAQKLFNDLFEDYSNALRPVEDTDKVLNVTLQITLSQIKDMDERNQILTAYLWIRQ 60
hα10   1    AEGRLALKLFRDLFANYTSALRPVADTDQTLNVTLEVTLSQIIDMDERNQVLTLYLWIRQ 60

rα1    59   QWVDYNLKWNPDDYGGVKKIHIPSEKIWRPDVVLYNNADGDFAIVKFTKVLLDYTGHITW 118
hα3    59   IWNDYKLKWNPSDYGGAEFMRVPAQKIWKPDIVLYNNAVGDFQVDDKTKALLKYTGEVTW 118
hα4    59   EWHDYKLRWDPADYENVTSIRIPSELIWRPDIVLYNNADGDFAVTHLTKAHLFHDGRVQW 118
hα7    59   SWTDHYLQWNVSEYPGVKTVRFPDGQIWKPDILLYNSADERFDATFHTNVLVNSSGHCQY 118
hα9    61   IWHDAYLTWDRDQYDGLDSIRIPSDLVWRPDIVLYNKADDESSEPVNTNVVLRYDGLITW 120
hα10   61   EWTDAYLRWDPNAYGGLDAIRIPSSLVWRPDIVLYNKADAQPPGSASTNVVLRHDGAVRW 120

rα1    119  TPPAIFKSYCEIIVTHFPFDEQNCSMKLGTWTYDGSVVAINPESDQPDLSNFMESGEWVI 178
hα3    119  IPPAIFKSSCKIDVTYFPFDYQNCTMKFGSWSYDKAKIDLVLIGSSMNLKDYWESGEWAI 178
hα4    119  TPPAIYKSSCSIDVTFFPFDQQNCTMKFGSWTYDKAKIDLVNMHSRVDQLDFWESGEWVI 178
hα7    119  LPPGIFKSSCYIDVRWFPFDVQHCKLKFGSWSYGGWSLDLQ--MQEADISGYIPNGEWDL 176
hα9    121  DAPAITKSSCVVDVTYFPFDNQQCNLTFGSWTYNGNQVDIFNALDSGDLSDFIEDVEWEV 180
hα10   121  DAPAITRSSCRVDVAAFPFDAQHCGLTFGSWTHGGHQLDVRPRGAAASLADFVENVEWRV 180

rα1    179  KEARGWKHWVFYSCCPNTPYLDITYHFVMQRLPLYFIVN 217
hα3    179  IKAPGYKHDIKYNCCE-EIYPDITYSLYIRRLPLFYTIN 216
hα4    179  VDAVGTYNTRKYECCA-EIYPDITYAFVIRRLPLFYTIN 216
hα7    177  VGIPGKRSERFYECCK-EPYPDVTFTVTMRRRTLYYGLN 214
hα9    181  HGMPAVKNVISYGCCS-EPYPDVTFTLLLKRRSSFYIVN 218
hα10   181  LGMPARRRVLTYGCCS-EPYPDVTFTLLLRRRAAAYVCN 218
```

```
rβ1    1    SEAEGQLLKKLFSD--YDSSVRPAQEVGDRVGVSIGLTLAQLISLNEKDEEMSTKVYLDL 58
hβ2    1    TDTEERLVEHLLDPSRYNKLIRPATNGSELVTVQLMVSLAQLISVHEREQIMTTNVWLTQ 60
hβ4    1    ANAEEKLMDDLLNKTRYNNLIRPATSSSQLISIKLQLSLAQLISVNEREQIMTTNVWLKQ 60
mε     1    KNEELSLYHHLFDN--YDPECRPVRRPEDTVTITLKVTLTNLISLNEKEETLTTSVWIGI 58
rδ     1    LNEEQRLIQHLFEEKGYNKELRPVARKEDIVDVALSLTLSNLISLKEVEETLTTNVWIDH 60

rβ1    59   EWTDYRLSWDPAEHDGIESLRVTAESVWLPDVVLLNNNDGNFDVALDINVVVSFEGSVRW 118
hβ2    61   EWEDYRLTWKPEEFDNMKKVRLPSKHIWLPDVVLYNNADGMYEVSFYSNAVVSYDGSIFW 120
hβ4    61   EWTDYRLTWNSSRYEGVNILRIPAKRIWLPDIVLYNNADGTYEVSVYTNLIVRSNGSVLW 120
mε     59   DWHDYRLNYSKDDFAGVGILRVPSEHVWLPEIVLENNIDGQFGVAYDSNVLVYEGGYVSW 118
rδ     61   AWIDSRLQWNANEFGNITVLRLPSDMVWLPEIVLENNNDGSFQISYACNVLVSDSGHVTW 120

rβ1    119  QPPGLYRSSCSIQVTYFPFDWQNCTMVFSSYSYDSSEVSLKTGPDPDGQ---ERQEIYIH 175
hβ2    121  LPPAIYKSACKIEVKHFPFDQQNCTMKFRSWTYDRTEIDLVLKSE------------VAS 168
hβ4    121  LPPAIYKSACKIEVKYFPFDQQNCTLKFRSWTYDHTEIDMVLMTP------------TAS 168
mε     119  LPPAIYRSTCAVEVTYFPFDWQNCSLIFRSQTYNAEEVEFIFAVDDDGN---TINKIDID 175
rδ     121  LPPAIFRSSCPISVTYFPFDWQNCSLKFSSLKYTAKEIRLSLKQEEEDNRSYPIEWIIID 180

rβ1    176  EGTFIENGQWEIIHKPSRLIHLPGDRRGGKEGHREEVIFYLIIRRKPLFYLVN 228
hβ2    169  LDDFTPSGEWDIVALPGRRNENPDDS------TYVDITYDFIIRRKPLFYTIN 215
hβ4    169  MDDFTPSGEWDIVALPGRRTVNPQDP------SYVDVTYDFIIKRKPLFYTIN 215
mε     176  TAAFTENGEWAIDYCPGMIRRYEGGSTEGP--GETDVIYTLIIRRKPLFYVIN 226
rδ     181  PEGFTENGEWEIVHRAAKVNVDPSVPMDST--NHQDVTFYLIIRRKPLFYIIN 231
```

Figure S2: Sequence alignment of the extracellular domain of rat (r), human (h) and mouse (m) nAChR subunits studied in this work. Fully conserved sequences are highlighted in dark gray, and partly conserved in light gray. The sequence of the N-terminal α-helix is underlined in α4 and β2 subunits. Two additional acidic residues, Asp2 and Asp13 in the N-terminal α-helix of β2 in comparison to β4 subunit, are colored red. Asp13 is more likely to be interacting with the Arg residues of NTD (see Figure 1b in the main text). The acidic residues in hα9, mε, and rδ subunit corresponding to Asp13 of β2 subunit are also colored red.
